# Geometric Accuracy Evaluation of High-Resolution Satellite Images Based on Xianning Test Field

**DOI:** 10.3390/s18072121

**Published:** 2018-07-02

**Authors:** Xiongwei Zheng, Qi Huang, Jingjing Wang, Taoyang Wang, Guo Zhang

**Affiliations:** 1 China Aero Geophysical Survey & Remote Sensing Center for Land and Resources, Beijing 100083, China; zheng_xiongwei@163.com; 2 School of Remote Sensing and Information Engineering, Wuhan University, Wuhan 430079, China; qihuang@whu.edu.cn; 3 Shandong Institute of Geological Surveying and Mapping, Shandong 250011, China; skdwangjing@126.com; 4 State Key Laboratory of Information Engineering in Surveying, Mapping and Remote Sensing, Wuhan University, Wuhan 430079, China; guozhang@whu.edu.cn

**Keywords:** high resolution satellite images, test field, geometrical performance, evaluation, accuracy

## Abstract

The evaluation of geometric accuracy of high-resolution satellite images (HRSIs) has been increasingly recognized in recent years. The traditional approach is to verify each satellite individually. It is difficult to directly compare the difference in their accuracy. In order to evaluate geometric accuracy for multiple satellite images based on the same ground control benchmark, a reliable test field in Xianning (China) was utilized for geometric accuracy validation of HRSIs. Our research team has obtained multiple HRSIs in the Xianning test field, such as SPOT-6, Pleaides, ALOS, ZY-3 and TH-1. In addition, ground control points (GCPs) were acquired with GPS by field surveying, which were used to select the significant feature area on the images. We assess the orientation accuracy of the HRSIs with the single image and stereo models. Within this study, the geometrical performance of multiple HRSIs was analyzed in detail, and the results of orientation are shown and discussed. As a result, it is feasible and necessary to establish such a geometric verification field to evaluate the geometric quality of multiple HRSIs.

## 1. Introduction

During the last decade, with the improvement of the resolution of HRSIs (Table 1), whose geometric positioning capabilities have also been gradually enhanced, the use of satellite images to produce large-scale topographic maps has become possible. Due to the distinctive feature of high-precision positioning of HRSIs, as well as the stringent requirements of high accuracy and high reliability in mapping, establishing a high stability and long-term continuous-operation scientific testing base for remote sensing and mapping will have significance and practical value.

Many research institutions, as well as experts and scholars, have done a lot of investigation and research work for the geometric calibration and validation of sensors. The Modular Optoelectronic Multispectral Scanner (MOMS-2P) was developed by DLR (Deutsches Zentrum für Luft- und Raumfahrt). The DLR updated the calibration data combined with photogrammetric bundle adjustment using an adapted functional model for the reconstruction of the interior orientation. In addition, it also compares the results of geometric laboratory calibration. The calibration field is located in the southern part of Germany and Austria [1,2]. Fraser et al. use different model to process the IKONOS images. The results can yield 3D object-point determination with an accuracy of 0.5 m in plane and 0.7 m in height. The GCPs are collected at road roundabouts or other distinct features conducive to high-precision measurement in both the imagery and on the ground [3]. Tadono et al. describe the updated plans for sensor calibration and product validation of the Panchromatic Remote-sensing Instrument for Stereo Mapping (PRISM), which is to fly on the Advanced Land Observing Satellite (ALOS). They not only evaluate the geometric accuracy of PRISM data, but also validate the derived DEM [4]. The Finnish Geodetic Institute has maintained a permanent test field for geometric, radiometric, and spatial resolution calibration and the testing of high-resolution airborne and satellite imaging systems in Sjökulla since 1994. The experience of nearly 10 years has shown that the use of gravel, combined with appropriate markers as the control objectives of the test field, can effectively eliminate the effects of seasonal and weather changes. It is also durable and able to guarantee the stability and consistency of the test field [5]. Meguro and Fraser evaluated a stereo pair of pansharpened GeoEye-1 Basic images covering the Tsukuba Test Field in Japan, which contains more than 100 precisely surveyed and image-identifiable GCPs. They indicated that the direct georeferencing accuracy is 2 m (CE90, the circular error of above 90% points) in plane and 3 m (LE90, the line error of above 90% points) in height. The use of a few GCPs improved the geopositioning accuracy to around 0.35 m (0.7 pixel) in plane and 0.7 m (1.4 pixel) in height [6]. John Dolloff, et al. use the Metric Information Network (MIN) method to process all 50 WorldView-1 stereo pairs. Statistics based on 101 ICPs (Independent Check Points) show that the positioning result is 0.5 m in plane and 0.3 m in height [7]. G. Agugiaro et al. evaluated the accuracy of GeoEye-1 and WorldView-2 by control and check data of the Trento test field in Italy. Also, 3D information extraction of the images was mentioned. For reference and validation, a DSM (Digital Surface Model) from airborne LiDAR acquisition is used as a comparison [8]. H. Topan and D. Maktav validated that different variations of point distribution and EOP configuration were preferred, achieving georeferencing accuracies of ~±1 m and ~±5 m at control and check points, respectively [9]. Wang et al. validated that ZY-3 can be used for the generation of cartographic maps at the 1:50,000 scale and for revision and updates of 1:25,000 scale maps [10,11,12,13,14]. By detecting and eliminating various kinds of geometric processing error, including equipment installation error, attitude and orbit measurement error, camera distortion, time synchronization errors and other errors, Li et al. found that the geometric orientation accuracy of Chinese satellite images could be improved to be better than 1.5 pixel, which is higher than the designed accuracy [15]. Tian et al. showed that more accurate and reliable assessment results can be obtained by choosing the appropriate evaluation method of geometric positioning accuracy [16].

Worldwide, scholars have done a lot of geometric accuracy verification work with multiple types of HRSIs. In previous research processes, the evaluation area and evaluation method of each satellite are different. The geometric positioning accuracy of each satellite could only be compared by related reports and papers. A unified test field is hoped to be established and a unified evaluation method is used to evaluate the geometric positioning accuracy of different satellites. The Xianning test field can meet this demand. Data collection, processing, and evaluation processes will become standardized with the Xianning test field, and the accuracy of the evaluation results can be compared more simply and intuitively.

Moreover, the designers of the test field will generally consider satellite geometry calibration and validation work in the same field. The internal and external orientation parameters of the satellite will be updated by the calibration. However, it requires a time lag after calibration in order to ensure the reliability of the accuracy. Therefore, a special validation test field is necessary only for accuracy evaluation using the ICPs (Independent Check Points) in the test field. In addition, this validation test field is different from the calibration test field. The main purpose of the validation test field is to verify the geopositioning accuracy and object recognition capabilities of the standard HRSI, and also to provide a reference for the direct application of the HRSI. The Xianning test field is such a test field for accuracy validation of HRSI or other remote sensing data.

## 2. Test Field Area and Data Sources

### 2.1. The Test Field

The test area is located in Xianning, a city of about 2,880,000 inhabitants in the south of Hubei province, central of China. The test field is situated in plain and hilly topography and the elevation ranges from 20 m to 400 m. This topographic feature is representative of flat and hilly land in China. The test field varies from urban areas with residential, industrial and commercial buildings at different sizes and heights, to agricultural or forested areas, and steep rocky surfaces, therefore offering a heterogeneous landscape in terms of geometrical complexity, land use and cover. Also, it needs convenient transportation and to be away from crowded areas in order to ensure that no deformation of the surface features will take place over time.

**Figure 1:**
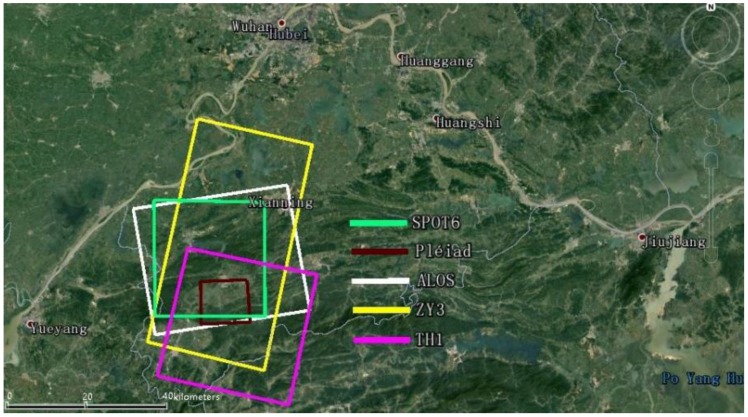
Thumbnail of multiple HRSIs coverage.

The area with the GCPs in the test field should contain at least two standard scenes to be able to make the most of the width of whole satellite images within the scope of the test field. Therefore, it is believed that the width of test field should be about 120 km (based on the value of double the current maximum width of the HRSIs in Table 1). A length of the test field of about 100 km along the track direction would be most appropriate; also double the standard length of one HRSI. Currently, the Xianning test field can meet the requirements of the width and length of most mainstream HRSIs. Thumbnail of multiple HRSIs coverage is shown in Figure 1.

### 2.2. The GCPs

**Figure 2:**
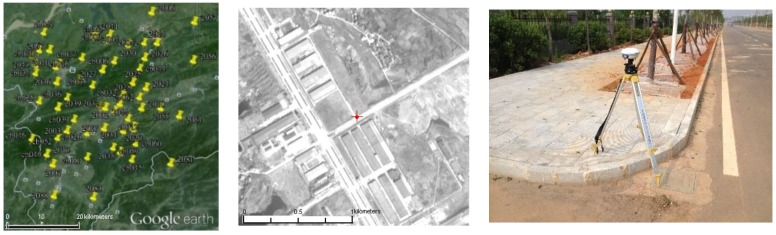
Distribution map of GCPs in the Xianning test filed.

Precision ground control data is the guarantee of geometric accuracy validation of HRSIs. Our group has done related work about GCPs laid in the Xianning region. A total of 118 GCPs are located in the test field as shown in Figure 2. Natural and artificial object feature points are chosen as GCPs by a static GPS field survey. The control points can be evenly distributed in each image, and the accuracy is about 10 cm, which is able to fully meet the needs of control and check points for all types of HRSIs.

### 2.3. Multiple HRSIs Data

Our team acquired 5 kinds of HRSIs within the range of the Xianning test filed. The HRSI data are summarized in Table 2, and include:

- **Pleiades**: The processing level is primary product. Primary product is the processing level closest to the natural image acquired by the sensor. This product restores perfect collections: the sensor is placed in rectilinear geometry, and the image is clear of all radiometric distortion. RPCs (Rational Polynomial Coefficients) and the sensor model are provided with the product. The data is a standard image product which includes nadir-forward-backward panchromatic images.
- **SPOT-6**: The processing level is primary product. The image is corrected for radiometric and sensor distortions, using internal calibration parameters, ephemeris and attitude measurements. RPCs and the sensor model are provided with the product. The data is a standard image product which includes nadir-forward-backward panchromatic images.
- **ALOS PRISM**: The processing level is 1B1 product. On the basis of Level 1A, the data with radiometric correction and added absolute calibration coefficient. RPCs and the sensor model are provided with the product. The data is a standard image product which includes nadir-forward-backward panchromatic images.
- **ZY-3**: The processing level is Sensor Corrected, i.e., the images are radiometrically and sensor corrected, but not projected to a plane using a map projection or datum, thus keeping the original acquisition geometry. The images were provided with RPCs. The data is a long strip image product which includes nadir-forward-backward panchromatic images [17].
- **TH-1**: the processing level is 1B; for each image, the RPCs were provided. The images are radiometrically and sensor corrected. The data is a standard image product which includes nadir-forward-backward panchromatic images. Other details about the characteristics of various satellite sensors are listed in Table 2.

## 3. General Geometric Processing Model of HRSIs

As seen in Section 2, all HRSIs are provided with RFM (Rational Function Model). Toutin and Teo’s study has shown that the result of satellite image orientation based on the RFM is almost as accurate as that based on the rigorous geometric model [18,19]. Additionally, the RFM has a simple form and leads to fast computations, so the orientation selects the RFM as the geometric model. The RFM describes the relations between the image point coordinates $\left(r_{n},c_{n}\right)$ and the ground point coordinates (*X_n_, Y_n_, Z_n_*), which have the following general form [20]:

(1) $$\left\{ \begin{array}{l} r_{n}=\frac{p_{1}(X_{n},Y_{n},Z_{n})}{p_{2}(X_{n},Y_{n},Z_{n})}\\ c_{n}=\frac{p_{3}(X_{n},Y_{n},Z_{n})}{p_{4}(X_{n},Y_{n},Z_{n})} \end{array}\right.$$

where (*r_n_*, *c_n_*) are measured line and sample coordinates of the *n*th image point, corresponding to the ground point with the object space coordinates (*X_n_, Y_n_, Z_n_*), which are the variables of a polynomial *p_i_* (*i* = 1, 2, 3, 4), whose degree should not exceed three. For example, the form of the polynomial *p*_i_ is

(2) $$\begin{array}{l} p_{i}=a_{i}{}_{0}+a_{i}{}_{1}Z+a_{i}{}_{2}Y+a_{i}{}_{3}X+a_{i}{}_{4}ZY+a_{i}{}_{5}ZX+a_{i}{}_{6}YX+a_{i}{}_{7}Z^{2}+a_{i}{}_{8}Y^{2}+a_{i}{}_{9}X^{2}+a_{i}{}_{10}ZYX+\\ \;a_{i}{}_{11}Z^{2}Y+a_{i}{}_{12}Z^{2}X+a_{i}{}_{13}Y^{2}Z+a_{i}{}_{14}Y^{2}X+a_{i}{}_{15}ZX^{2}+a_{i}{}_{16}YX^{2}+a_{i}{}_{17}Z^{3}+a_{i}{}_{18}Y^{3}+a_{i}{}_{19}X^{3} \end{array}$$

where $a_{ij}(i=1,2,3,4,\;j=0,\;1,\;\cdots ,\;19)$ are rational polynomial coefficients [20].

Furthermore, bias compensation is needed. Previous studies have shown that the affine model combined with RFM can eliminate the systematic errors in the image points, which improves the geometry processing accuracy [11]. Therefore, we modify the relationship between the image coordinates (*x*, *y*) and the coordinates (*X*, *Y*, *Z*) according to Formula (3) [21].

(3) $$\{\begin{array}{c} x+a_{0}+a_{1}x+a_{2}y=\frac{P_{1}\left(X,Y,Z\right)}{P_{2}\left(X,Y,Z\right)}\\ y+b_{0}+b_{1}x+b_{2}y=\frac{P_{3}\left(X,Y,Z\right)}{P_{4}\left(X,Y,Z\right)} \end{array}$$

The affine transformation parameters $\left(a_{0},a_{1},a_{2},b_{0},b_{1},b_{2}\right)$ are set as orientation parameters. It can be solved with a small number of GCPs.

## 4. Validation of Geometric Accuracy for HRSIs

The test is expected to demonstrate to what extent direct georeferencing and sensor orientation are accurate and efficient methods for the determination of the exterior orientation parameters for topographic mapping. Currently, such assessments are performed through the validation technique known as the Hold-Out Validation (HOV) method [22]. It is also known as test sample estimation. According to this, the data set (known ground points) is partitioned into two subsets: the first one is used in the orientation model (GCPs—Ground Control Points) and the second one is used to validate the model itself (ICPs—Independent Check Points).

### 4.1. Orientation Accuracy with Single Image

The image orientation determines the relation between the object and the image coordinates, which is dependent on the image product and the imaging mode. So at least for reliability, GCPs are required. The image orientation can be based on a geometric reconstruction of the imaging geometry, depending upon the available information. The direct sensor orientation may be available, too, as a sensor-oriented RPC. Like the geometric reconstruction, this can be improved by GCPs, named and also bias corrected [21]. 

**Figure 3:**
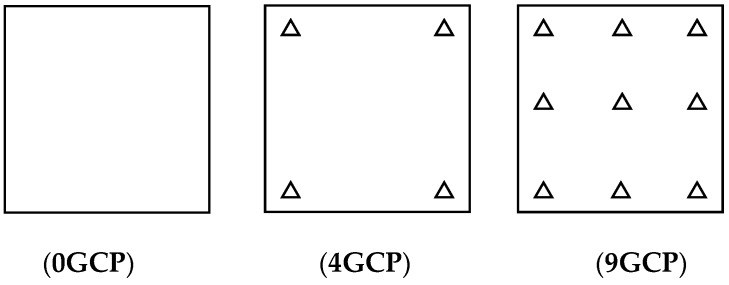
GCP scheme.

**Figure 4:**
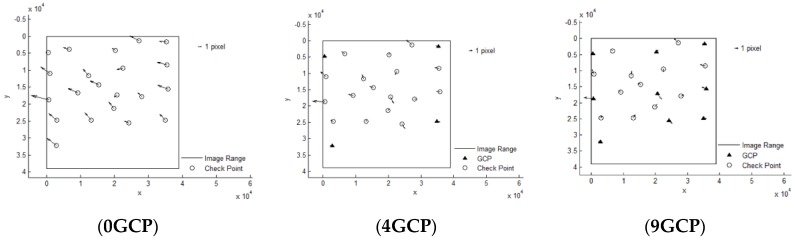
Residual distributions of check points of orientation for Pleaides.

**Figure 5:**
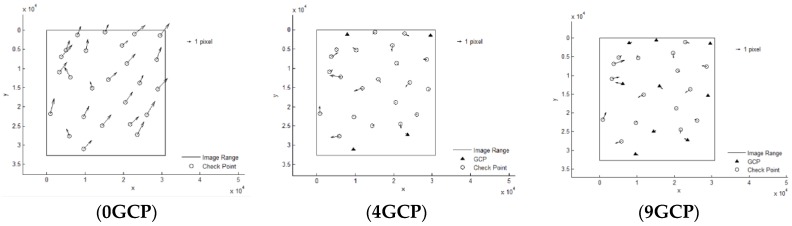
Residual distributions of check points of orientation for SPOT6.

**Figure 6:**
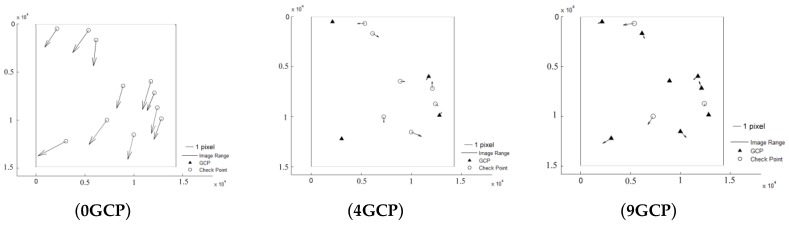
Residual distributions of check points of orientation for ALOS.

**Figure 7:**
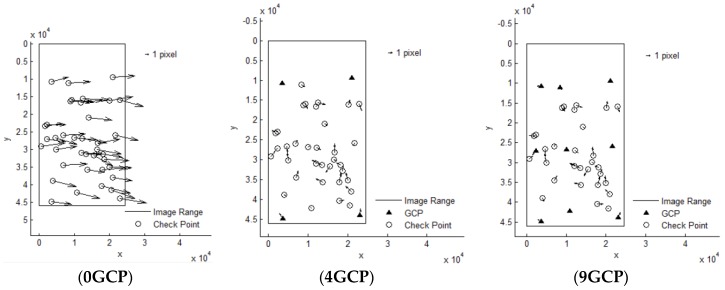
Residual distributions of check points of orientation for ZY-3.

**Figure 8:**
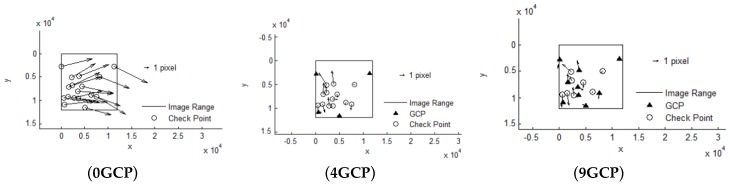
Residual distributions of check points of orientation for TH-1.

The orientation accuracy was analyzed and verified based different schemes of laid GCPs, as follows in Table 3:

It can be seen that Pleaides and SPOT6 performed with the highest accuracy without GCPs, almost reaching the 3 pixel level, from Table 3. The results of ZY-3 and TH-1 are almost the same, reaching about 6 pixels. When 4 GCPs were laid in the four corners, ZY-3, SPOT6 and Pleaides all reached the 1.5 pixel level or better. However, residuals of some points in the TH-1 image were still large after orientation with GCPs. The interior geometric accuracy of TH-1 is poor, as can be seen from the residuals distributions in Figure 3. More GCPs were added, but no more obvious changes occurred. Therefore, the scheme with 4 GCPs located in each corner is recommended for HRSI orientation. Residual distribution figures are shown below in Figure 4, Figure 5, Figure 6, Figure 7 and Figure 8:

### 4.2. Orientation Accuracy of Block Adjustment with Tri-Stereo Images

All these HRSIs are able to constitute a three linear array stereo model with nadir-forward-backward images. In consideration of the multi-covered image data with redundant observations information, block adjustment was carried out with and without GCPs to evaluate the orientation accuracy. Conclusive results are as follows:

From Table 4 it can be seen that in the situation of no GCP, Pleaides reached an accuracy of 0.860 m in plane and 2.654 m in height, which is really unbelievable, although the 0.5 m GSD (Ground Sampling Distance) of Pleaides is the highest among the HRSIs. SPOT6 reached an accuracy of 5.336 m in plane and 4.595 m in height, and also has a superior performance in geometric accuracy without GCP. The block adjustment accuracy without GCP of Pleaides and SPOT6 meet the requirements for 1:50,000 Topographic maps. However, ALOS, ZY-3 and TH-1 cannot reach that level. 

From Table 4 it can also be seen that in the situation with GCPs, when four GCPs were laid in the four corners, the block adjustment accuracy of ZY-3 reaches 2.653 m in plane and 1.858 m in height, and ALOS reaches 2.900 m in plane and 1.363 m in height. Compared to the accuracy without GCP, this accuracy is improved a lot, reaching the level of SPOT6, with the accuracy in height being even better. Pleaides still has the best performance, while TH-1 has the worst performance, reaching 17.152 m in plane and 7.467 m in height, although its resolution of 5 m is the lowest. Under the condition of setting the GCPs in the four corners, without consideration of artificial pricking points, the accuracy almost reaches the best level for single image orientation and for block adjustment. The accuracy undergoes no more changes, even when more GCPs are added. Therefore, four GCPs laid in the corners is a good layout scheme, which is recommended.

From another point of view, if a satellite image cannot achieve an accuracy of 1–2 pixels with four GCPs laid, it can be shown that the internal geometric distortion has not been eliminated before the generation of standard image products. For satellite images without internal geometric distortion, the four GCPs laid can eliminate most of the errors and achieve high-precision positioning.

## 5. Conclusions

Different from the traditional methods of verification, the paper has embarked on a unified test investigating sensor orientation and describes the processing carried out on ZY-3, TH-1, ALOS, SPOT6 and Pleaides in a geometric accuracy test field, instead of verifying them separately. All the results are compared under conditions both without and with GCPs, and whether in orientation with single image or in block adjustment. The performance of Pleaides is the best. SPOT6, ALOS and ZY-3 are almost comparable, although the ground resolution of SPOT6 is slightly higher than that of ZY-3. The performance of TH-1 is a little worse. A layout scheme of four GCPs laid in the corners is recommended, and can be used for geometric precision processing and evaluation of HRSIs.

Also, the test field set up in Xianning is presented with the aim of investigating spaceborne optical imagery. The reason for choosing the Xianing area as the test field is explained in detail, and the function of the Xianning test field is also illustrated for the geometric accuracy validation of HRSIs. The test field will undoubtedly be important both for development, analysis, and simulation of platforms and sensors in the future. In addition, verification work of the HRSI data obtained will continue to be carried out.

## Figures and Tables

**Table 1:** High-resolution optical satellite system. PAN: panchromatic, MS: multispectral, SWIR: Shortwave infrared.

Satellite	Nation	Launch Date	Bands	Spatial Resolution/m	Width/km
IKONOS	America	24 September 1999	PAN/MS	1/4	11
QuickBird	America	18 October 2001	PAN/MS	0.61/2.44	16.5
SPOT-5	France	4 May 2002	PAN/MS/SWIR	2.5/10/20	60
SPOT-6	France	9 September 2012	PAN/MS	1.5/6.0	60
CBERS-02B	China	21 October 2003	PAN/MS	2.36/20	27
Cartosat-1	India	5 May 2005	PAN	2.5 (forward), 2.2 (backward)	26
ALOS	Japan	24 January 2006	PAN/MS	2.5/10	35/70
EROS-B	Israel	25 April 2006	PAN	0.7	14
Cartosat-2	India	10 January 2007	PAN	<1	10
WorldView-1	America	18 September 2007	PAN	0.5	17.6
GeoEye-1	America	6 September 2008	PAN/MS	0.41/1.65	15.2
WorldView-2	America	8 October 2009	PAN/MS	0.46/1.84	16.4
WorldView-3	America	13 August 2014	PAN/MS/SWIR	0.31/1.24	13.2
ZY3	China	9 January 2012	PAN/MS	2.1 (nadir)/3.5 (forward, backward)/5.8	52
TH-1	China	24 August 2010	PAN/MS	2 (HR)/5 (forward, backward, nadir)/10	60
Pléiades	France	17 December 2011	PAN/MS	0.5/2.0	20

**Table 2:** Characteristics of satellite imagery in the Xianning test field. PAN: panchromatic, GSD: Ground Sample Distance.

**Satellite**	Pléiades	SPOT-6	ALOS PRISM	ZY3	TH1
**Country**	France	France	Japan	China	China
**Imaging Time**	12 July 2013	11 July 2013	27 September 2006	16 February 2013	8 June 2013
**Spatial Resolution**	Panchromatic: 0.5 m Multispectral: 2 m	Panchromatic: 1.5 m Multispectral: 6 m	Panchromatic: 2.5 m Multispectral: 10 m	Panchromatic: 2.1 m/3.5 m Multispectral: 5.8 m	Panchromatic: 2 m Multispectral: 10 m Three line array: 5 m
**Orbit Height**	694 km	695 km	692 km	506 km	500 km
**Nominal Positioning Accuracy**	8.5 m	35 m	50 m	50 m	200 m
**Width of Image**	20 km	60 km	35 km	50 km	60 km
**Side Pendulum Angle**	Standard: ±30° Max: ±47°	Standard: ±30° Max: ±45°	±44°	±32°	±32°
**Focal Length**	12.905 m	6.023 m	1.939 m	1.7 m	2.187 m
**Pixel Size**	13 µm	13 µm	7 µm	7 µm	8 µm
**Data Level**	primary product	Standard product	Level 1B1	SC	Level 1B
**Base to Height Ratio**	0.3	0.4	1	0.88	1
**Stereoscopic Mode**	Along-track/Across-track	Along-track/Across-track	Along-track/Across-track	Along-track	Along-track
**Imaging Mode**	Three line array/pushbroom	Three line array/pushbroom	Three line array/pushbroom	Three line array/pushbroom	Three line array/pushbroom

**Table 3:** Orientation accuracy of HRSIs.

Satellite	Number of GCP	Number of ICP	RMSE of GCP (Pixels)			RMSE of ICP (Pixels)		
			*x*	*y*	Plane	*x*	*y*	Plane
Pleaides	0	21	-	-	-	2.300	1.344	2.664
	4	17	0.768	0.082	0.772	0.929	1.322	1.591
	9	12	1.370	0.700	1.538	0.760	0.891	1.172
SPOT6	0	26	-	-	-	1.655	2.556	3.045
	4	22	0.381	0.166	0.415	1.121	0.821	1.390
	9	17	0.876	0.357	0.946	1.217	0.817	1.466
ALOS	0	11	-	-	-	2.193	3.943	4.511
	4	7	0.300	0.404	0.503	0.972	0.703	1.199
	9	2	0.768	0.560	0.951	0.337	0.698	0.775
ZY-3	0	38	-	-	-	6.342	1.180	6.451
	4	34	0.362	0.175	0.402	0.872	1.160	1.451
	9	29	0.546	0.479	0.726	0.853	1.123	1.410
TH-1	0	17	-	-	-	6.413	2.158	6.766
	4	13	0.444	0.049	0.447	0.591	1.622	1.726
	9	8	0.493	1.100	1.205	0.639	1.467	1.600

**Table 4:** Accuracy of block adjustment for HRSIs.

Satellite	Number of GCP	Number of ICP	RMSE of GCP (m)				RMSE of ICP (m)			
			*x*	*y*	Plane	Height	*x*	*y*	Plane	Height
Pleaides	0	21	-	-	-	-	0.588	0.628	0.860	2.654
	4	17	0.123	0.044	0.130	1.977	0.510	0.445	0.677	1.505
	9	12	0.574	0.340	0.667	1.530	0.403	0.395	0.564	1.377
SPOT6	0	26	-	-	-	-	3.362	4.144	5.336	4.595
	4	22	0.121	0.329	0.351	0.917	1.842	1.169	2.182	2.129
	9	17	1.231	0.480	1.321	2.209	2.006	1.309	2.396	2.294
ALOS	0	16	-	-	-	-	8.677	31.588	32.758	11.832
	4	12	1.639	1.489	2.214	0.717	1.655	2.381	2.900	1.363
	9	7	1.263	1.393	1.880	1.326	1.681	3.208	3.621	2.125
ZY-3	0	38	-	-	-	-	12.818	4.263	13.508	11.528
	4	34	0.030	0.289	0.291	0.259	1.565	2.142	2.653	1.858
	9	29	0.355	0.257	0.438	1.442	1.640	2.356	2.870	1.937
TH-1	0	32	-	-	-	-	29.095	15.089	32.775	12.228
	4	28	0.001	0.001	0.001	0.001	8.703	14.780	17.152	7.467
	9	23	0.687	4.306	4.361	1.900	4.770	8.674	9.899	5.028

## References

[1] Komus W., Lehner M., Blechinger F., Putz E. (1996). Geometric calibration of the stereoscopic CCD-line-scanner MOMS-2P. Int. Arch. Photogramm. Remote Sens..

[2] Komus W., Lehner M., Schroeder M. (2000). Geometric in-flight calibration of the stereoscopic CCD-line-scanner MOMS-2P. ISPRS J. Photogramm. Remote Sens..

[3] Fraser C., Hanley H., Yamakawa T. (2002). Three-Dimensional Geopositioning Accuracy of Ikonos Imagery. Photogramm. Rec..

[4] Tadono T., Shimada M., Watanabe M., Hashimoto T., Iwata T. (2004). Calibration and validation of PRISM onboard ALOS. Int. Arch. Photogramm. Remote Sens. Spat. Inf. Sci..

[5] Honkavaara E., Peltoniemi J., Ahokas E., Kuittinen R., Hyyppä J., Jaakkola J., Kaartinen H., Markelin L., Nurminen K., Suomalainen J. (2008). A Permanent Test Field for Digital Photogrammetric systems. Photogramm. Eng. Remote Sens..

[6] Meguro Y., Fraser C.S. (2010). Georeferencing accuracy of Geoeye-1 stereo imagery: Experiences in a Japanese test field. Int. Arch. Photogramm. Remote Sens. Spat. Inf. Sci..

[7] Dolloff J., Settergren R. Worldview-1 stereo extraction accuracy without MIN processing. Proceedings of the ASPRS 2010 Annual Conference.

[8] Agugiaro G., Poli D., Remondino F. (2012). Testfield Trento: Geometric evaluation of very high resolution satellite imagery. Int. Arch. Photogramm. Remote Sens. Spat. Inf. Sci..

[9] Topan H., Maktav D. (2014). Efficiency of Orientation Parameters on Georeferencing Accuracy of SPOT-5 HRG Level-1A Stereoimages. IEEE Trans. Geosci. Remote Sens..

[10] Wang T.Y., Zhang G., Li D.R., Tang X.M., Jiang Y.H., Pan H.B., Zhu X.Y., Fang C. (2014). Geometric accuracy validation for ZY-3 satellite imagery. IEEE Geosci. Remote Sens. Lett..

[11] Wang T., Zhang G., Li D., Tang X.M., Jiang Y.H., Pan H.B., Zhu X.Y. (2014). Planar Block Adjustment and Orthorectification of ZY-3 Satellite Images. Photogramm. Eng. Remote Sens..

[12] Zhang G., Wang T.Y., Li D., Tang X., Jiang Y.H., Huang W.C., Pan H. (2015). Block adjustment for satellite imagery based on the strip constraint. IEEE Trans. Geosci. Remote Sens..

[13] Pan H., Tao C., Zou Z. (2016). Precise georeferencing using the rigorous sensor model and rational function model for ZiYuan-3 strip scenes with minimum control. ISPRS J. Photogramm. Remote Sens..

[14] Pan H. (2017). Geolocation error tracking of ZY-3 three line cameras. ISPRS J. Photogramm. Remote Sens..

[15] Li D., Zhang G., Jiang Y., Shen X. (2016). Research on image geometric precision of domestic optical satellites. Spacecr. Eng..

[16] Tian G., Huang Q., He H., Xia Z. (2017). Analysis on Geometric Positioning Accuracy Evaluation of Remote Sensing Satellite Image. Spacecr. Recovery Remote Sens..

[17] Pan H., Zhang G., Tang X., Li D., Zhu X., Zhou P., Jiang Y. (2013). Basic Products of the ZiYuan-3 Satellite and Accuracy Evaluation. Photogramm. Eng. Remote Sens..

[18] Toutin T. (2006). Comparison of 3D physical and empirical models for generating DSMs from stereo HR images. Photogramm. Eng. Remote Sens..

[19] Teo T.-A. (2011). Bias compensation in a rigorous sensor model and rational function model for high-resolution satellite images. Photogramm. Eng. Remote Sens..

[20] Tao C.V., Hu Y. (2001). A comprehensive study of the rational function model for photogrammetric processing. Photogramm. Eng. Remote Sens..

[21] Fraser C.S., Hanley H.B. (2003). Bias compensation in rational functions for IKONOS satellite imagery. Photogramm. Eng. Remote Sens..

[22] Brovelli M.A., Mattia M., Fratarcangeli F., Giannone F., Realini E. (2008). Accuracy assessment of high resolution satellite imagery orientation by leave-one-out method. ISPRS J. Photogramm. Remote Sens..

